# Instruments to measure fear of COVID-19: a diagnostic systematic review

**DOI:** 10.1186/s12874-021-01262-5

**Published:** 2021-04-23

**Authors:** Ashley Elizabeth Muller, Jan Peter William Himmels, Stijn Van de Velde

**Affiliations:** Norwegian Institute of Public Health, PO Box 222 Skøyen, 0213 Oslo, Norway

**Keywords:** Fear, Psychometrics, Diagnostic accuracy, SARS-CoV-2

## Abstract

**Background:**

The COVID-19 pandemic has become a source of fear across the world. Measuring the level or significance of fear in different populations may help identify populations and areas in need of public health and education campaigns. We were interested in diagnostic tests developed to assess or diagnose COVID-19-related fear or phobia.

**Methods:**

We performed a systematic review of studies that examined instruments diagnosing or assessing fear or phobia of COVID-19 (PROSPERO registration: CRD42020197100). We utilized the Norwegian Institute of Public Health’s *Live map of covid-19 evidence*, a database of pre-screened and pre-categorized studies. The *Live map of covid-19 evidence* identified references published since 1 December 2019 in MEDLINE, Embase, and the Centers for Disease Control and Prevention. Following biweekly searches, two researchers independently categorized all studies according to topic (seven main topics, 52 subordinate topics), population (41 available groups), study design, and publication type. For this review, we assessed for eligibility all studies that had been categorized to the topic “*Experiences and perceptions, consequences; social, political, economic aspects”* as of 25 September 2020, in addition to hand-searching included studies’ reference lists. We meta-analyzed correlation coefficients of fear scores to the most common reference tests (self-reports of anxiety, depression, and stress), and reported additional concurrent validity to other reference tests such as specific phobias. We assessed study quality using the QUADAS-2 for the minority of studies that presented diagnostic accuracy statistics.

**Results:**

We found 18 studies that validated fear instruments. Fifteen validated the Fear of COVID-19 scale (FCV-19S). We found no studies that proposed a diagnosis of fear of COVID-19 or a threshold of significant/clinical versus non-significant/subclinical fear. Study quality was low, with the most common potential biases related to sampling strategy and un-blinded data analysis. The FSV-19S total score correlated strongly with severe phobia (*r* = 0.703, 95%CI 0.634–0.761) in one study, and moderately with anxiety in a meta-analysis.

**Conclusions:**

The accuracy of the FSV-19S needs to be measured further using fear-related reference instruments, and future studies need to provide cut-off scores and normative values. Further evaluation of the remaining three instruments is required.

**Supplementary Information:**

The online version contains supplementary material available at 10.1186/s12874-021-01262-5.

## Background

Fear is an emotional response to a threat as perceived by an individual, and considered a functional, adaptive, and transient response to stimuli, briefly resulting in physiological changes [1, 2]. Fear can become pathological when physiological changes are chronic instead of transient, when fear reactions are triggered in the absence of actual danger, and/or when an individual is unable to learn safety signals, that is, to inhibit fear reactions by understanding safety cues (see [3, 4]).

Infectious diseases are a particularly salient source of fear because they are transmissible, imminent, and invisible [2], and the COVID-19 pandemic has become a source of fear across the world. Schimmenti and colleagues [5] have suggested four dialectical elements of COVID-19 fear: fear of and for one’s body, as one is both a potential vector and victim; fear of and for others, also related to the tension of prescribed social distancing from important interpersonal relationship; fear of ignorance of the virus as well as knowledge, as information is required for protection but can also be overwhelming and anxiety-inducing; and fear of both personal action and inaction, related to the behavioral consequences of fear. More than four out of five respondents reported one of the respective fears in two large surveys that included 1421 Japanese workers [6] and 669 dental practitioners worldwide [7]. In a recent systematic review of the mental health impact of COVID-19, healthcare workers reported that fear tied to their professional responsibilities extended into their personal lives [8]. Fear of getting sick with COVID-19 at work because they were unable to protect themselves extended to fear of infecting family members at home. This review found that fear correlated with greater exposure risk, supporting the general understanding of fear to be an appropriate response to an external threat.

Several surveys have also demonstrated that those more directly impacted by the pandemic or those at risk of being personally impacted are more afraid, as seen in people currently laid off or furloughed [9], and in people at high risk due to comorbidities [6, 10]. Fear may have both positive consequences, such as greater adherence to infection prevention and control strategies, and negative consequences, such as avoiding health care services and settings. Harper et al. [11] recently reported a moderate positive correlation of fear to transmission-reducing behavior change such as hand-washing and social distancing (*r* = 0.31) in an international survey of 324 respondents. Karacin et al. [12] found that 14% of a Turkish center’s oncology patients cited COVID-19 fear as the reason they delayed their chemotherapy (by an average of 47 days), despite no COVID-19 cases among patients or staff during the data collection period. Patients’ and relatives’ fear of infection was the overwhelming reason hypothesized by staff at 227 of China’s 280 stroke hospitals – only half of which treated COVID-19 patients – to explain nationwide reductions in hospital admissions for thrombolysis/thrombectomy [13].

Individuals’ fear can amass into critical social and public health problems. Taylor and colleague’s early questionnaires reported that fear of COVID-19 was highly correlated with stigmatization of both healthcare workers and foreigners [14, 15]. These findings are an unfortunate reminder of Ebola outbreak research that pointed to fear-driven behavior with serious economic and social consequences, including stigmatization and discrimination of survivors [16–18]. Bali et al. [19] introduced the term “fearonomics” to describe these effects during Ebola. However, the threshold at which individuals’ fear transforms into population-level problems is unclear: do a certain amount of people need to experience pathological or phobic fear, or a larger amount of people experiencing subclinical fear? Does the position of people in a society experiencing fear – e.g. a minority of policymakers versus a majority of voters – influence when fears become problematic on a population level?

Measuring the level or significance of fear in different populations may help identify populations and areas in need of public health and education campaigns. Several validated instruments are available to serve as gold standard reference tests to diagnosis phobic and non-phobic fears related to illness, such as the Illness Attitudes Scales [20], the Fear Survey Schedule III [21], and the Perceived Vulnerability to Disease [22]. These gold standard reference tests and others have been used in the development of fear scales of Ebola [23] and swine flu [24].

Many studies measuring fear of COVID-19 have used unvalidated instruments, although the Fear of COVID-19 Scale [25] is increasingly used. It is imperative that fear of COVID-19 be measured appropriately. This systematic review examines scales used to assess or diagnosis fear of COVID-19.

## Method

We conducted a systematic review according to our protocol registered in PROSPERO (CRD42020197100), and following Cochrane’s guidelines for diagnostic accuracy tests.

### Inclusion criteria

We included all studies that identified themselves as diagnostic accuracy or validation studies, and that measured the accuracy or validated the psychometric properties of any instrument measuring fear of COVID-19. Widening the selection of study designs beyond diagnostic accuracy studies was based on Umemneku Chikere and colleagues’ recent systematic review of diagnostic accuracy tests conducted in the absence of a gold standard reference test, which found the use of validation studies to comprise one of four methodological alternatives [26]. We had no exclusion criteria related to population, intervention, comparator, outcome, or language; however, Chinese-language studies were not identified in our search strategy.

### Literature search and article selection

We identified relevant studies by searching the Norwegian Institute of Public Health’s (NIPH’s) publicly available *Live map of covid-19 evidence* (https://www.fhi.no/en/qk/systematic-reviews-hta/map/) on 25 September 2020. This map is one of several evidence maps and living evidence databases that attempt to enable faster identification of relevant publications with primary data. Not only has the speed with which covid-19 publications proceed through the peer-review and publication processes increased exponentially, even when compared to previous pandemics [27], the majority of publications on COVID-19 do not in fact present data [28]. Researchers and evidence synthesizers face significant challenges in identifying relevant publications, beginning simply by filtering out those lacking empirical data. Numerous initiatives have begun that use human effort, machine learning, or a combination, to identify relevant studies, categorize them according to intervention or population group, and in some cases extract and analyze data *before* a systematic review has been ordered, so that researchers will be positioned to rapidly produce high-quality evidence syntheses on a variety of topics as soon as policymakers request them. Some of the larger initiatives include the COVID-NMA project, focused on continuously updating analyses of randomized trials [29]; Epistemonikos’ LOVE database, which categorizes studies according to “type of question”, suitable for systematic reviews [30]; and the EPPI Centre’s living map, containing 11 heterogenous and mutually exclusive categories such as vaccine development, genetics, and mental health impacts [31].

NIPH’s *Live map of covid-19 evidence* is one of the most granular evidence maps, and all studies are categorized according to topic (seven main topics, 52 subordinate topics), population (41 available groups), study design, and publication type, by two researchers independently. The map’s protocol describes the methodology in detail [32]. The search strategy of the *Live map of covid-19 evidence* has developed dynamically since March 2020, and is updated online [32]. The *Live map of covid-19 evidence* first searched for references on 12 March 2020, and identified references published since 1 December 2019 in MEDLINE, Embase, Centers for Disease Control and Prevention, and the Center for Evidence-Based Medicine. EPPI Centre at University College London has conducted the majority of searches (MEDLINE and Embase) and screening from May 2020 and onwards [31].

For this systematic review, we identified references categorized to the topic “*Experiences and perceptions, consequences; social, political, economic aspects”.* One researcher screened all identified references specifically for the inclusion criteria for this systematic review, and read in full-text those containing the words *fear*, phobi*, diagnos*, accuracy, psychometric*, validation, or *scale*, in title/abstract. We also hand-searched included studies’ reference lists. On the search day, the map’s website stated that all relevant studies identified in database searches on or before 4 May 2020 were categorized, along with systematic reviews, health technology assessments, and randomized and non-randomized controlled trials up until 24 August 2020.

### Data extraction and methodological quality assessment

We developed a data extraction form to collect data on country, target group and participants (age, gender, COVID-19 characteristics and socioeconomic characteristics as reported by study author), instrument description, methods of accuracy/validity assessment (such as reference tests), and outcomes. One researcher extracted data, and a second researcher checked extraction for accuracy.

We planned on using the QUADAS-2 [33] for diagnostic accuracy studies to assess methodological quality. Only three studies [34–36] tested a fear instrument against a dichotomous reference test and could therefore be assessed as diagnostic accuracy studies; the remainder measured convergent validity to continuous scores of other patient-reported outcomes. We assessed only these three studies using the QUADAS-2.

### Data presentation and analyses

While we intended to conduct a summary ROC analysis to assess sensitivity and specificity of fear instruments, only one of the included studies produced an area under the curve score. In lieu of a summary ROC analysis, we reported each study’s validation assessments using classical test theory or Rasch analysis, namely measures of internal consistency reliability, factor structure, and goodness-of-fit tests (Additional file 1). We meta-analyzed correlation coefficients between total fear instrument scores and other reference tests’ total scores. We reported the correlation of fear to other constructs reported by at least one study, and calculated 95% confidence intervals. We conducted all analyses in STATA v.16 [37] using the metan command.

## Results

### Results of the literature search

**Fig. 1:**
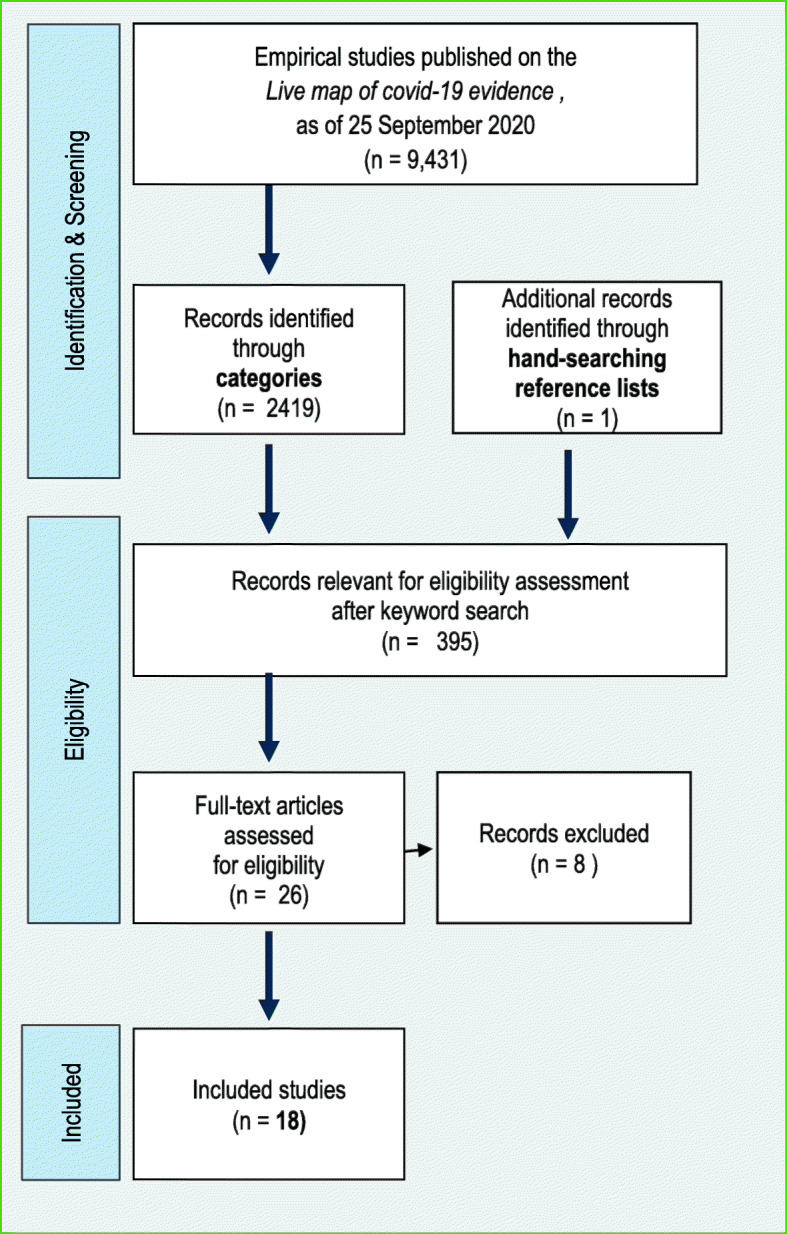
Live evidence map flow diagram of study inclusion*.* As of 25 September 2020, the Live map of covid-19 evidence project in cooperation with EPPI Centre had categorized 9431 studies. Twenty-six were assessed in full-text for this review, and 18 included

As of 25 September 2020, the *Live map of covid-19 evidence* project in cooperation with EPPI Centre had screened 56,977 studies for COVID-19 relevance, and categorized 9431 studies with empirical data, either primary, secondary, or modelled. Within the studies categorized to the topic *Experiences*, we identified 394 with a relevant keyword in title/abstract. One additional study was identified through hand-searching studies’ references lists. After assessing these 395 studies for eligibility, 18 met our inclusion criteria [25, 35, 36, 38–52] (Fig. 1).

### Description of studies

Eighteen studies were included, with a large geographic spread. Three studies took place in Turkey, two studies each in China, Peru, and Israel, and one each in Iran, Bangladesh, Italy, Saudi Arabia, Vietnam, Unites States, and Greece; one study sampled participants from both Belarus and Russia. All studies but one were online questionnaires that recruited via social media or used other convenience sampling methods. Most studies targeted the general population; four sampled among university students/graduates, one recruited participants working at public health departments, with no further description of sampling methods [45], and another recruited patients receiving psychiatric treatment [41]. No studies were conducted specifically among healthcare personnel. Sample sizes ranged from 228 to 8550 participants (Table 1).

**Table 1:** Characteristics of the 18 included studies

Study	Instrument	Setting, target group, and participant description	Language	Test accuracy or validity methods^**a**^
Ahorsu et al. [25]	FCV-19S (Fear of COVID-19 Scale) – development paper	Iran *N* = 717 general population Mean age: 31.25 years (SD 12.68), Female: 42.0% COVID-19 characteristics: NR Socioeconomic: 8.9 years of education (SD 4.1)	Iranian, with English translation provided	Convergent validity (HADS Anxiety, HADS Depression, PVDS). Internal consistency reliability.
Alyami et al. [38]	FCV-19S	Saudi Arabia *N* = 639 general population Mean age: 34.75 years (SD 11.80) Female: 42.1% COVID-19 characteristics: NR Socioeconomic: 70% university qualification; 50.2% employed, 15.6% unemployed, 27.4% student	Arabic	Convergent validity (HADS total score, HADS Anxiety, HADS Depression). Internal consistency reliability.
Arpaci et al. [39]	COVID-19 Phobia Scale (C19P-S) – development paper	Turkey Study 1: *N* = 1250 general population Mean age: 37.53 years (SD = 16.94) Female: 61.2% COVID-19 characteristics: 0.4% positive Socioeconomic: 1.8% high, 21.3% middle-high, 57.5% middle, 14.6% low. Study 2: *N* = 2143 general population Mean age: 39.66 years (SD 16.87) Female: 60–3% COVID-19 characteristics: 0.5% positive Socioeconomic: 1.5% high, 20.2% middle-high, 60.1% middle, 12.7% middle-low, 12.7% low	Turkish, with English translation provided	Reference test: COVID-19 infection vs not. Internal consistency reliability.
Bitan et al. [40]	FCV-19S	Israel *N* = 649 Mean age: NR Female: 84.8% COVID-19 characteristics: 52.9% unemployed during lockdown, 58.3% main career during COVID-19, 18.0% in risk group for COVID-19 mortality and 77.0% not in risk group, 4.1% direct contact with COVID-19 patient, 0.6% COVID-19 death in family Socioeconomic: 33.2% above average, 45.6% below average, 21.1% average	Hebrew	Convergent validity (DASS-21), internal consistency reliability.
Chang et al. [41]	FCV-19S	Taiwan *N* = 400 adults receiving inpatient or outpatient treatment for psychiatric disorder Mean age: 46.91 years (SD 10.92) Female: 44.5% COVID-19 status: NR Socioeconomic: Mean education years 11.31 (SD 2.98)	Chinese	Internal consistency reliability.
Feng et al. [42]	Scale of COVID-19 related psychological distress in healthy public (CORPD) – development paper	China *N* = 652 uninfected healthy people Mean age: NR Female: 67.3% COVID-19 status: NR Socioeconomic: 82.8% had college degree or above, 56.4% had a monthly income of more than 4000 yuan	Chinese	Convergent validity (SCL-90), internal consistency reliability:
Haktanir et al. [43]	FCV-19S	Turkey *N* = 668 general population Mean age: NR Female: 72.0% COVID-19 status: NR Socioeconomic: 31.4% high, 61.4% middle, 7% low	Turkish	Convergent validity (BRS), internal consistency reliability.
Huarcaya-Victoria et al. [44]	FCV-19S	Peru *N* = 832 general population Mean age: 38.37 years (SD 12.75) Female: 65.6% COVID-19 status: 68.4% without any symptoms of COVID-19, 21.0% with one symptom, 6.5% with two symptoms, 3.5% with three symptoms, 0.6% with four or more symptoms Socioeconomic: 76.4% with university education, 66.9% with formal employment	Spanish	Convergent validity (GAD-7, PHQ-9, IES-R), internal consistency reliability:
Mejia et al. [45]	Fear Perception and Magnitude of the Issue (MED-COVID-19) – development paper	Peru *N* = about 400 public employees Mean age: NR Female: NR COVID-19 status: NR Socioeconomic: NR	Spanish, Portuguese	Internal consistency reliability
Nguyen et al. [35]	FCV-19S	Vietnam *N* = 5423 university students Mean age: 22.0 years (SD 2.0) Female: 52.0% COVID-19 status: 18.9% with suspected symptoms, 81.0% without suspected symptoms Socioeconomic: 53.9% with very or fairly easy ability to pay for medication, 46.0% very or fairly difficult ablity to pay	Vietnamese	Reference test: AUC to distinguish GAD ≥8. Internal consistency reliability:
Pang et al. [46]	FCV-19S	Malaysia *N* = 228 university students Mean age: NR Female: 71.1% COVID-19 status: NR Socioeconomic: 1.8% doctoral degree, 5.7% master degree, 56.1% bachelor degree, 26.8% diploma, 9.6% high school	Malay	Convergent validity (DASS-21), internal consistency reliability.
Perz et al. [47]	FCV-19S	United States *N* = 237 university students Mean age: 30.3 years (SD 10.2) Female: 73.0% COVID-19 status: 29% know someone with COVID-19 symptoms Socioeconomic: 73% negative financial impact by COVID-19 or response	English	Convergent validity (GAD-7), internal consistency validity
Reznik et al. [48]	FCV-19S	Belarus and Russia *N* = 850 university students/ graduates Mean age: 34.8 (SD 13.0) Female: 73.2% COVID-19 status: NR Socioeconomic: 65.4% university graduate, 28.4% university student, 6.2% primary or secondary school education	Russian	internal consistency reliability
Sakib et al. [49]	FCV-19S	Bangladesh *N* = 8550 general population Mean age: 26.5 years (SD 9.1) Female: 44.0% COVID-19 status: NR Socioeconomic: 82.0% educated at tertiary level, 59.6% student, 3.7% unemployed	Bangal	Convergent validity (PHQ-9), internal consistency reliability
Satici et al. [50]	FCV-19S	Turkey *N* = 1304 general population Mean age: NR Female: 70.3% COVID-19 status: 1.7% symptoms, 12.9% partial symptoms, 85.4% no symptoms Socioeconomic: 10.0% graduate degree, 79.8% bachelor degree, 3.8% associate degree, 5.0% high school, 1.4 less than high school	Turkish	Convergent validity (DASS-21, SWLS), internal consistency reliability
Soraci et al. [51]	FCV-19S	Italy *N* = 249 general population Mean age: 34.50 years (SD 12.21) Female: 92% COVID-19 status: NR Socioeconomic: 58.7% university-level degree, 39% high school degree, 2.4% lower-level educational degree	Italian	Convergent validity (HADS, SMSP-A), internal consistency reliability
Tsipropoulou et al. [52]	FCV-19S	Greece *N* = 2970 general population Mean age: NR Female: 72.5% COVID-19 status: NR Socioeconomic: 45.4% university degree, 29.8% high school degree, 1.5% less than high school. 8.9% health care provider.	Greek	Convergent validity (PHQ-9, GAD-7), internal consistency reliability
Zolotov et al. [36]	FCV-19S	Israel *N* = 370 university students Mean age: 25.2 (SD 3.1) Female: 78.1% COVID-19 status: NR Socioeconomic: NR	Hebrew	Reference test: convergent validity to single items regarding COVID-19-related depression, anxiety, nervousness, loneliness, and exhaustion. Internal consistency reliability:

*NR* Not reported. Other instruments: *HADS* Hospital Anxiety and Depression Scale, *SMSP-A* Severity Measure for Specific Phobia— Adult, PHQ-9:, DASS.21:, *GAD-7* Generalized Anxiety Disorder scale, *BRS* Brief Resilience Scale, *SCL-90* Symptom Checklist-90, *PVDS* Perceived Vulnerability to Disease Scale

**Fig. 2:**
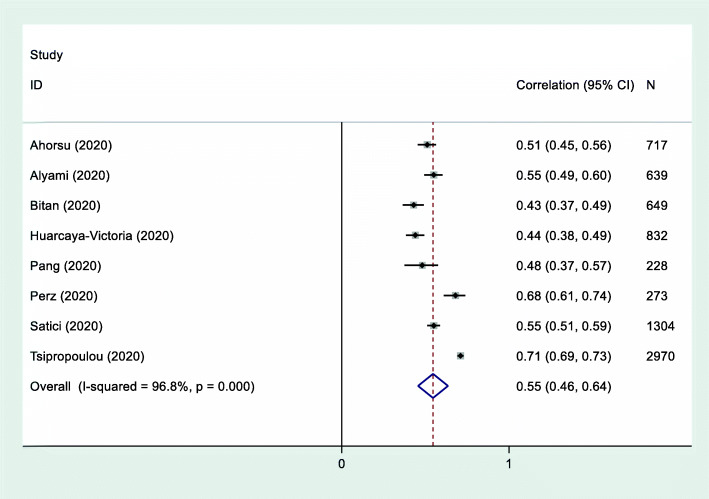
Correlation between FSV-19S and anxiety. Eight studies measured the correlation of fear scores to anxiety

**Fig. 3:**
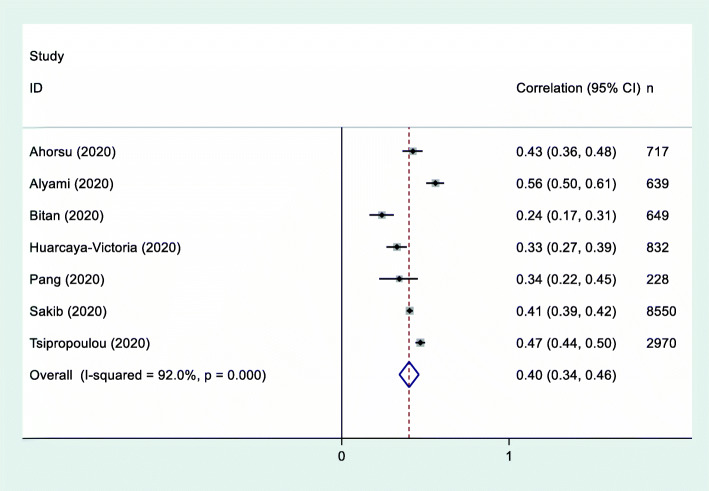
Correlation between FSV-19S and depression. Seven studies measured the correlation of fear scores to depression

**Fig. 4:**
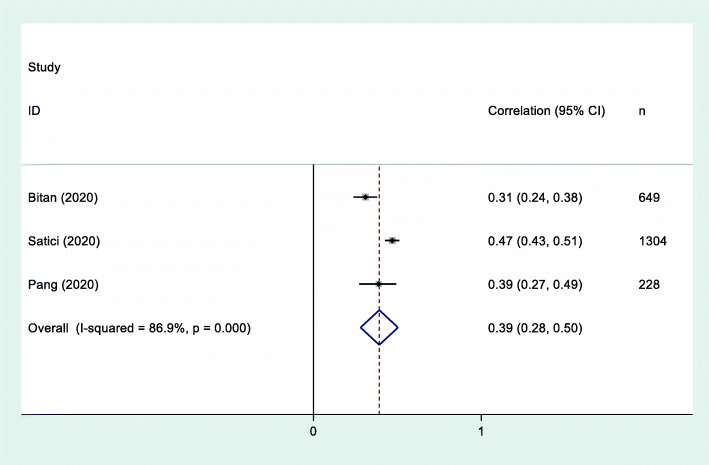
Correlation between FSV-19S and stress. Three studies measured the correlation of fear scores to stress

**Fig. 5:**
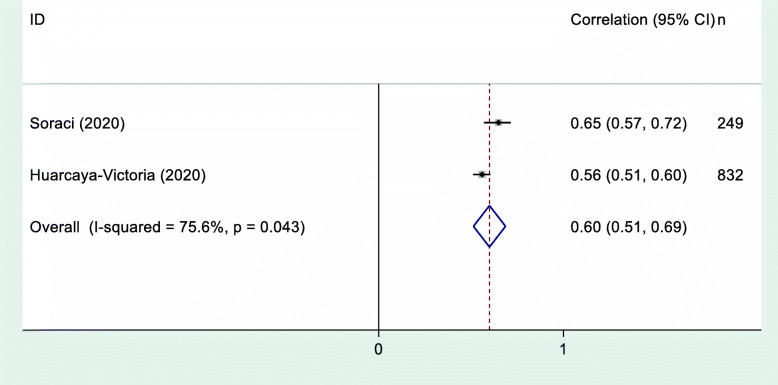
Correlation between FSV-19S and HADS total score. Two studies measured the correlation of fear scores to distress

^a^Convergent validity is displayed in forest plots in Figs. 2, 3, 4 and 5. Other validity assessments are in Additional file 1

### Instruments

The eighteen included studies validated four instruments. No study validated more than one instrument.

#### Fear of COVID-19 Scale (FCV-19S)

Fifteen studies validated the Fear of COVID-19 Scale (FCV-19S), including the original development study. This seven-item instrument was developed by Ahorsu et al. [25]. The authors first identified thirty general instruments on fear through a literature review, pooled and de-duplicated relevant items, solicited expert input to further reduce the amount of items, piloted the resulting ten-item instrument among 46 non-clinical participants for feasibility, administered it among a larger sample (*N* = 717), and finally, removed three items following classical test theory analysis. Respondents indicate their agreement with each question, as for example “I am afraid of losing my life because of coronavirus”, on a 1–5 Likert-type scale from “strongly disagree” to “strongly agree”. All answers are summed to produce a total score, from 7 to 35, with higher scores indicating more fear.

While the original authors and eleven subsequent studies concluded the FCV-19S was unidimensional, Bitan et al. [40] found a two-factor fit of emotional fear and symptomatic expressions of fear. Huarcaya-Victorial et al. [44] found a bifactor model, in which all seven items load onto one general factor and onto one of two subordinate factors of emotional fear and symptomatic expressions, as in Bitan et al. Zolotov et al. reported weak fit indices for a one-factor and a two-factor model, but did not report the items comprising the two factors. With the exception of Zolotov et al., all studies utilizing classical test theory or Rasch analysis reported a stable factor structure, and adequate fit indices (see Additional file 1).

Most studies that used reference tests used validated and patient-reported screening measures of symptom severity. No studies used fear diagnoses as reference tests. Nguyen et al. [35] reported the area under the curve and 95% confidence intervals to predict clinically significant anxiety on the Generalized Anxiety Disorder Screener (scores ≥8). The area under the curve was 0.63 (0.60–0.66); meaning FCV-19S scores correctly distinguished participants with clinically significant anxiety from clinically non-significant anxiety in 63% of cases. Zolotov et al.’s [36] reference tests were self-reports of how much more depressed, anxious, nervous, exhausted, or lonely participants felt in the past month during the COVID-19 pandemic; results are presented under the sub-headings of anxiety and depression.

Thirteen studies tested convergent/concurrent validity to reference tests with continuous scores as a validation method, with all reporting significant bivariate correlations between the FCV-19 total score (and each sub-score, in the case of Bitan et al. [40]; not reported) and the various psychological constructs tested (Fig. 2).

Across nine studies, greater anxiety correlated with greater fear on the FSV-19S (*r* = 0.55, CI 0.46–0.64), as displayed in Fig. 2. Zolotov et al. [36] measured anxiety dichotomously, and found that the total FSV-10S score distinguished between self-reports of feeling more anxious as a result of COVID-19 in the past month, versus not.

Figure 3 shows that greater depression also correlated with greater fear (*r* = 0.4, CI 0.34–0.46). The total score distinguished between participants who reported feeling more depressed as a result of the pandemic in the past month and those who did not report feeling more depressed, reported by Zolotov et al. [36].

Stress was measured in three studies (Fig. 4), and more stress correlated with more fear (*r* = 0.39, CI 0.28–0.50).

The Hospital Anxiety and Depression Scale total score, a measure of general distress, was correlated with the FSV-19S (*r* = 0.60, CI 0.51–0.69) (Fig. 5).

Each meta-analysis contained a very large amount of unexplained heterogeneity, a strong suggestion that the studies’ populations, contexts, or methods were not similar enough to combine. It is therefore useful for readers to visually examine individual studies’ results in each forest plot.

Concurrent validity was suggested according to significant correlations between the FSV-19S total score and perceived infectability from the Perceived Vulnerability to Disease Scale (*r* = 0.483, CI 0.425–0.537), and germ aversion from the same scale (*r* = 0.459, CI 0.400–0.512) in Ahorsu et al.’s development study [25]. Severe phobia also correlated positively with FSV-19S total score (*r* = 0.703, CI 0.634–0.761) [51].

More resilience was associated with less fear (*r* = 0.32, CI −.386 to −.250) in Haktanir et al. [43], and greater life satisfaction was associated with less fear (*r* = − 0.200, CI −.252 to −.147) in Satici et al. [50].

#### Fear perception and magnitude of the issue (MED-COVID-19) scale

The MED-COVID-19 was developed by a group of experts, piloted, and administered to approximately 400 public employees in Peru [45]. Only one validation study was identified for this instrument, which measured both the extent of fear and fear sources, such as media, healthcare providers, and family/friends. Three factors were uncovered within twelve items through Rasch analysis. The first factor contained items relating to the perception that media sources were exaggerating COVID-19, the second factor related to magnitude of fear from media, and the third factor to both perception and magnitude of fear arising from healthcare providers, family, and friends. The validation study did not assess the MED-COVID-19 against a reference test, assess concurrent validity, or report subscale scores or total scores.

#### Scale of COVID-19 Related Psychological Distress in the healthy public (CORPD)

The Scale of COVID-19 [42] related psychological distress in the healthy public (CORPD), consists of two factors: fear/anxiety and suspicion. Both dimensions had satisfactory internal reliability; α = 0.742 and 0.869, respectively) The CORPD was developed through structured interviews with eleven uninfected, healthy individuals, expert input following the Delphi method, and a pilot test in China. The total score correlated moderately with anxiety, measured on the SCL-90 subscale (γ = 0.31, CI 0.24–0.38).

#### COVID-19 Phobia Scale (C19P-S)

The COVID-19 Phobia Scale [39] is based on a pool of 102 DSM-5 diagnostic criteria for phobia, adapted to COVID-19. Key criteria were excessive or unreasonable persistent fear, immediate anxiety provoked by exposure which may manifest as panic, recognition by the individual that the fear is excessive or unreasonable, avoidance of exposure, and anxiety that significantly interferes with normal routines if exposure cannot be avoided. A panel of physicians and psychometricians rated each item according to relevance; 70 items were identified and surveyed among 1250 participants. Exploratory factor analysis suggested retaining 20 items, and confirmatory factor analysis was conducted after a separate sample of 2143 participants answered these 20 items. The total score was a sum of four subscales: psychological, psychosomatic, economic, and social. The only reference test administered was COVID-19 infection, and the total C19P-S score as well as each factor score distinguished between infected and non-infected participants.

### Methodological quality assessment

Table 2 displays results of the QUADAS-2 assessment for the only three studies that could be assessed as diagnostic accuracy studies. Nguyen et al. tested the discrimination of the FSV-19 against clinically significant anxiety, Zolotov et al. tested the discrimination of the FSV-19 against anxiety specifically resulting from COVID-19, and Arpaci et al. tested the discrimination of the C19P-S against COVID-19 infection or non-infection. Each of these studies had low overall methodological quality. The patient selection domain had the most risks of biases across studies; participants were often convenience samples and/or recruited through social media, reducing the likelihood that they were representative of the general populations that most studies aimed to sample from. All three were cross-sectional studies conducted online. No studies performed analyses that kept the results of the fear instrument blinded from the results of the reference test.

**Table 2:**
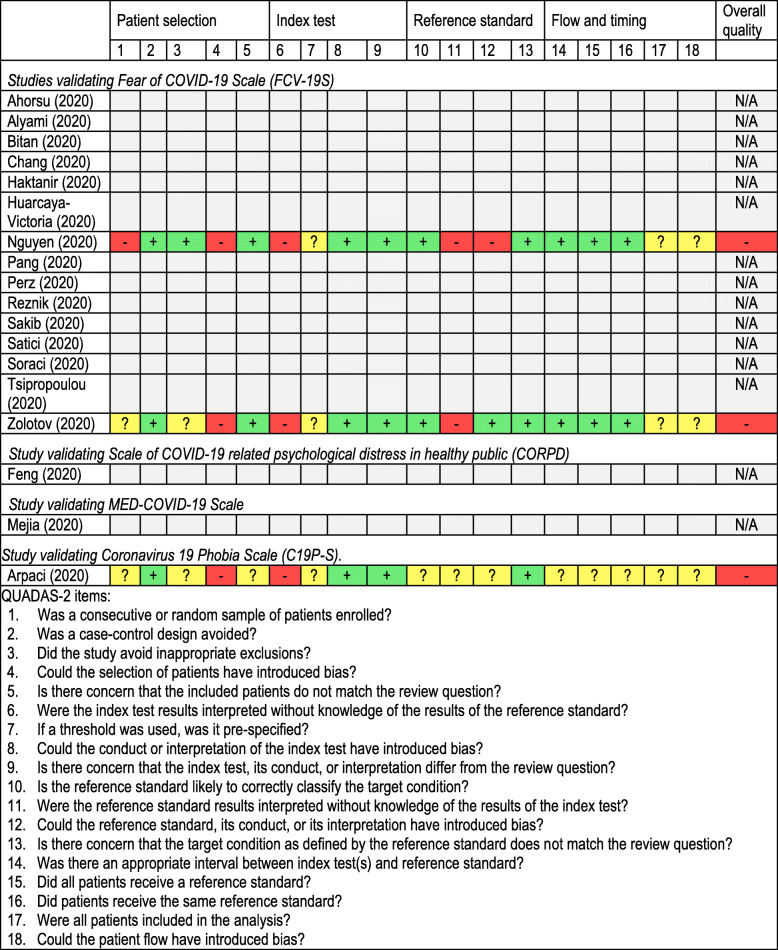
Methodological quality assessment with QUADAS-2

## Discussion

This systematic review searched for diagnostic accuracy or instrument validation studies of fear of COVID-19 instruments on 25 September 2020, and identified eighteen studies involving participants from seventeen countries. Fifteen studies validated the seven-item Fear of COVID-19 scale (FCV-19S). Based on scientific interest across the world, this scale has been translated to 13 languages. The overall score, indicating greater fear, correlated moderately to anxiety in a meta-analysis of correlation coefficients (*r* = 0.55, 95%CI 0.46–0.64). One study from Peru validated the Fear Perception and Magnitude of the Issue (MED-COVID-19) scale, one study from China validated the Scale of COVID-19 related psychological distress in healthy public (CORPD), and one study from Turkey validated the Coronavirus 19 Phobia Scale (C19P-S).

All but seven studies used anxiety as a reference test. Only two [25, 51] used disease fear or phobia-specific reference tests, and it is noteworthy that the highest correlation coefficient was between a self-reported measure of specific phobia (*r* = 0.703 CI 0.634–0.761) and fear (FSV-19S total score). The only study that reported a standard diagnostic accuracy metric used FSV-19S to discriminate among clinically significant vs non-significant self-reported anxiety scores, which was possible in only 63% of cases: a poor level of accuracy [53]. Accuracy may have been higher had a fear, not anxiety, reference test been used. It is likely that sampling procedures and data collection modes – mainly convenience samples recruited through online social media – necessitated administration of simpler and non-diagnostic reference tests. The high amounts of unexplained heterogeneity in meta-analyses may be partially accounted for by the variety of reference tests. Heterogeneity may also be explained by an unstable factor structure. While twelve studies reported the FSV-19 to be unidimensional, one study found a two-factor fit and another a bi-factor fit; further research needs to explore dimensionality. As Schimmenti and colleagues [54] and Mertens and colleagues [55] have noted, fear may be more than pathological. Socioeconomic and interpersonal aspects of fear may be distinct factors or mediators of physiological manifestations of fear, and it is crucial that fear instruments are able to stably measure these dimensions.

The authors of included studies were able to quickly administer and collect data by choosing online convenience sampling, but therefore introduced potential sampling bias that reduced methodological quality in the three studies assessed with the QUADAS-2. The evidence so far supports the continued exploration of the FSV-19S based on a high correlation to severe phobia, and a moderate correlation to anxiety. To increase trustworthiness in these results, future studies should confirm this among non-selected samples, and should utilize fear-specific reference tests. The available evidence on the additional three instruments is not robust enough for recommending their use. The C19P-S was recently validated among an American sample in a forthcoming article (personal communication with I. Arpaci); depending on the findings, it may be a more appropriate instrument that uses the DSM-5-specific phobia criteria to measure COVID-19 fear.

While fear is understood to be adaptive and transient, and an appropriate response to a threat, Ebola research has emphasized the negative effects of disease-related fear on the community and international level [56]. Individual fear of infection and fear resulting from witnessing disease progression and death can turn into “a cyclical pattern of fear” on the community level (p.211), in which normal community interactions are disrupted because individuals loose trust in health services and reproduce stigma against infected individuals and survivors. In the current pandemic, discrimination and hate crimes against Asians have already been reported in North America [57] and Europe [58, 59], a worrying reminder of the stigma and discrimination that Africans reported globally during the 2013/2014 Ebola outbreak [56]. Fear of COVID-19 is increasingly being reported in systematic reviews related to mental health as a distinct outcome [60] or an identified risk factor for mental health problems [61]. With methodologically strong instruments, fear of COVID-19 can be measured and populations with more fear identified to receive public education and public health campaigns.

### Strengths and weaknesses

This review is the first that we know of to systematically search for and assess diagnostic accuracy or other validation studies of fear of COVID-19 instruments. Our quality assessment of studies should help other researchers in the evidence synthesis process, if they choose to use methodological quality in their inclusion criteria. An additional methodological strength is our utilization of the publicly available *Live map of covid-19 evidence*, one of the first reviews to do so (others include Muller at al. [8], as well as a diagnostic accuracy review that used the map as one of multiple sources [62]). By using this map, we quickly identified 394 studies that had already been categorized to our topic of interest, without having to search in academic databases and screen again. Although this timely approach has increased the availability of overarching research, it includes the risk of missing relevant studies missing if they were not grouped to the relevant categories. We did not use any of the methodological shortcuts recently reported in a survey of rapid diagnostic review study authors [63].

## Conclusion

At least four instruments have been developed to measure fear of COVID-19, ten months after the pandemic began. Most studies are assessing the validity of instruments against other patient-reported mental health problems, rather than measuring diagnostic accuracy against fear-related reference tests. The Fear of COVID-19 scale (FCV-19S) instrument has already been translated to 13 languages, and our included studies reported convergent validity to phobia, disease-related fear, and anxiety. Future studies should assess diagnostic accuracy against validated fear-specific reference tests.

## Supplementary Information

**Additional file 1: Table 1.** Validity outcomes.

## Data Availability

Data may be available by the authors upon request.

## Abbreviations

COVID-19: Coronavirus disease 2019
CORPD: Scale of COVID-19 Related Psychological Distress in the Healthy Public
DSM: Diagnostic and Statistical Manual of Mental Disorders
FCV-19S: Fear of COVID-19 scale
MED-COVID-19: Fear Perception and Magnitude of the Issue scale
