# Supplementary material for: Instruments to measure fear of COVID-19: a diagnostic systematic review
Source: BMC Med Res Methodol. 2021 Apr 23;21:82. doi: 10.1186/s12874-021-01262-5 (PMC8064424; doi:10.1186/s12874-021-01262-5)
Supplement: Supplementary file 1 — Additional file 1: Table 1. Validity outcomes. [file 12874_2021_1262_MOESM1_ESM.docx]

**Additional file**

Additional file 1: Table 1: Validity outcomes

| **Study** | **Instrument** | **Validity outcomes from classical test theory and Rasch analysis** |
| --- | --- | --- |
| Ahorsu et al.(23) | FCV-19S (Fear of COVID-19 Scale) – development paper | Cronbach’s α =.82; test-retest reliability (ICC) = 0.72; Item separation reliability from Rasch = 0.99; infit MnSq values were between 0.80 and 1.26; outfit MnSq values were between 0.84 and 1.25. |
| Alyami et al.(34) | FCV-19S | Cronbach’s α= .88. In a confirmatory factor analysis, a model allowing correlation between error variances of two items had adequate model fit: CFI = 0.995, RMSEA = 0.059, and SRMR = 0.024. |
| Arpaci et al.(35) | COVID-19 Phobia Scale (C19P-S) – development paper | Four-factor structure of psychological, psychosomatic, economic, and social. Cronbach’s α per factor ranged 0.85 to 0.90. Confirmatory factor analysis indicated adequate model fit: χ2 (df=125) = 446.930, χ2/df = 3.575, p < .001, GFI = 0.979, NFI = 0.981, IFI = 0.986, TLI = 0.981, CFI = 0.986, and RMSEA = 0.035 (90% CI 0.031, 0.038). |
| Bitan et al.(36) | FCV-19S | Exploratory factor analysis: two-factor model explained 65.76% of the total variance, cronbach’s α = 0.77 for Factor 1 and 0.80 for Factor 2. |
| Chang et al.(37) | FCV-19S | Cronbach’s α = 0.93. Confirmatory factor analysis: χ2 (df)/p value = 31.64 (14)/0.005, CFI = 0.994, TLI = 0.992, RMSEA= 0.056 (90% CI 0.030, 0.082), SRMR = 0.057 |
| Feng et al.(38) | Scale of COVID-19 related psychological distress in healthy public (CORPD) – development paper | Cronbach’s α of Anxiety & fear dimension = 0.742, α of Suspicion dimension = 0.869. Split-Half reliability test showed that the Guttman Split-Half coefficient of the CORPD was 0.907, the Guttman Split-Half coefficient of the Anxiety & fear dimension was 0.705, and 0.858 for the Suspicion dimension. |
| Haktanir et al.(39) | FCV-19S | Confirmatory factor analysis: Final model paired error terms between items 3 and 6, 3 and 7, and 6 and 7. χ2 (df) = 30.45 (2.77). RMSEA = 0.05, SRMR =0.03, AGFI =0.97, NFI =0.98, IFI =0.99, GFI =0.99, CFI =0.99, TLI =0.98. |
| Huarcaya-Victoria et al.(40) | FCV-19S | Confirmatory factor analysis. Bifactor model was adequate in all fit indices (CFI and TLI > 0.90 and RMSEA and SRMR < 0.08). Unidimensional and two-factor models both had inadequate RMSEA. |
| Mejia et al.(41) | Fear Perception and Magnitude of the Issue (MED-COVID-19) – development paper | Internal consistency reliability |
| Nguyen et al.(31) | FCV-19S | AUC predicting GAD ≥ 8 = 0.63 (95% CI 0.60–0.66).  Cronbach’s α = 0.90. In a factor analysis, all seven items strongly loaded on one component, and explained 62.15% of the variance. Item–scale convergent validity, rho (range)= 0.77 (0.66–0.84). |
| Pang et al.(42) | FCV-19S | Infit MnSq values were between 0.83 and 1.38, and outfit MnSq values were between 0.76 and 1.30. Cronbach’s α = 0.893, McDonald’s ω = 0.894. Test-retest reliability = 0.971, p<0.001. |
| Perz et al.(43) | FCV-19S | Cronbach’s α = 0.91. Corrected item-total correlations of each item ≥ 0.70. In an exploratory factor analysis, all items loaded onto one factor that explained 66% of variance. |
| Reznik et al.(44) | FCV-19S | internal consistency reliability: Principal components analysis confirmed two factors (items 1, 2, 4, and 5, explaining 47% of variance; and items 3, 6, and 7, explaining 51% of variance) |
| Sakib et al.(45) | FCV-19S | Cronbach’s α = 0.871. χ2 (df) = 554.75 (14), p<0.001. CFI = 0.964. TLW = 0.947. RMSEA: 0.071. CR: 0.89. Item separation reliability from Rasch: 1.00. |
| Satici et al.(46) | FCV-19S | Cronbach’s α = .847, McDonald’s ω = .849, λ6 = .844 CR= .842.  χ2(13, N=1304) =299.47, p < .05; SRMR= .061; GFI = .936; NFI = .912; IFI = .915; CFI = .915. |
| Soraci et al.(47) | FCV-19S | Cronbach’s α = 0.871. TLI = .99, CFI = .99, RMSEA= 0.069; SRMR= 0.047. One-factor structure with 74.31% explained common variance. |
| Tsipropoulou et al.(48) | FCV-19S | Cronbach’s α = 0.87; RMSEA= 0.11, CFI = 0.89, TLI = 0.83, SRMR= 0.06. |
| Zolotov et al.(32) | FCV-19S | Cronbach’s α = 0.842, McDonald’s ω = 0.852.  Unidimensional CFA fit indices: CFI (comparative fit index) = 0.837; TLI = 0.829; and RMSEA = 0.146. The two-dimensional CFA fit indices were as follows: CFI = 0.937; TLI = 0.865; and, RMSEA= 0.107. |
| GFI, NFI, and CFI ≥ .90 and SRMR ≤ .08 are considered an indication of acceptable fit (Kline 2015).  CFI: Comparative fit index; Cronbach’s α = Cronbach’s alpha; CR: composite reliability; CI: confidence interval; IFI: incremental fit index; λ6: Guttmann’s lambda; McDonald’s ω: McDonald’s omega; RMSEA: root mean square error of approximation; SRMR: standardized root mean square residual; TLI: Tucker-Lewis Index. | | |
